# A Rasch and factor analysis of the Functional Assessment of Cancer Therapy-General (FACT-G)

**DOI:** 10.1186/1477-7525-5-19

**Published:** 2007-04-20

**Authors:** Adam B Smith, Penny Wright, Peter J Selby, Galina Velikova

**Affiliations:** 1 Psychosocial & Clinical Practice Research Group, Cancer Research UK Clinical Centre, St. James's University Hospital, Leeds, UK

## Abstract

**Background:**

Although the Functional Assessment of Cancer Therapy – General questionnaire (FACT-G) has been validated few studies have explored the factor structure of the instrument, in particular using non-sample dependent measurement techniques, such as Rasch Models. Furthermore, few studies have explored the relationship between item fit to the Rasch Model and clinical utility. The aim of this study was to investigate the dimensionality and measurement properties of the FACT-G with Rasch Models and Factor analysis.

**Methods:**

A factor analysis and Rasch analysis (Partial Credit Model) was carried out on the FACT-G completed by a heterogeneous sample of cancer patients (n = 465). For the Rasch analysis item fit (infit mean squares ≥ 1.30), dimensionality and item invariance were assessed. The impact of removing misfitting items on the clinical utility of the subscales and FACT-G total scale was also assessed.

**Results:**

The factor analysis demonstrated a four factor structure of the FACT-G which broadly corresponded to the four subscales of the instrument. Internal consistency for these four scales was very good (Cronbach's alpha 0.72 – 0.85). The Rasch analysis demonstrated that each of the subscales and the FACT-G total scale had misfitting items (infit means square ≥ 1.30). All these scales with the exception of the Social & Family Well-being Scale (SFWB) were unidimensional. When misfitting items were removed, the effect sizes and the clinical utility of the instrument were maintained for the subscales and the total FACT-G scores.

**Conclusion:**

The results of the traditional factor analysis and Rasch analysis of the FACT-G broadly agreed. Caution should be exercised when utilising the Social & Family Well-being scale and further work is required to determine whether this scale is best represented by two factors. Additionally, removing misfitting items from scales should be performed alongside an assessment of the impact on clinical utility.

## Background

Quality of life assessment is increasingly being used in routine clinical practice in oncology [1,2]. Furthermore, the assessment process itself has now been shown to improve the clinical consultation and patient well-being [3].

The Functional Assessment of Cancer Therapy – General (FACT-G) is a widely used quality of life instrument for cancer patients. The questionnaire was originally developed using semi-structured interviews of patients and oncology professionals to generate instrument items [4]. A factor analysis of the original 28-item version of the instrument revealed five factors corresponding to: Physical Well-being, Social & Family Well-being, Emotional Well-being, Functional Well-being and relationship with doctor [4]. Psychometric analyses of the instrument demonstrated that Cronbach's alpha was high for the total scale (0.89) indicating high levels of reliability. Similarly, test-retest reliability coefficients ranged between 0.82 (Emotional Well-being and Relationship with doctor) to 0.88 (Physical Well-being).

There has been extensive development and validation of a number of site- and disease-specific modules for the FACT, including, for instance modules for anaemia and fatigue, colorectal, breast and lung cancer. Furthermore, some additional validation has also been carried out on the FACT-G, including for instance, evaluations of minimally important differences [5] and identification of differential item functioning [6]. Winstead-Fry and Schultz [7] conducted a validation study of the FACT-G (version 2) on a sample of 344 cancer patients living in rural areas (i.e. non-metropolitan) in the USA. The factor analysis of the scores – transformed to log-odds or logits – revealed the same five subscales. Furthermore, Cronbach's alpha levels were within the same range as reported by Cella et al. [4]. Kemmler et al. [8] investigated the structure of the FACT-G (version 2) using multidimensional scaling. This analysis revealed that most subscales, but particularly Physical and Social & Family Well-being, as well as the Relationship with doctors scales, demonstrated high levels of consistency with items from each subscale clustering together. Items from the Functional Well-being scale showed higher degrees of scatter, and there was an amount of overlap between Emotional and Functional Well-being.

More recently, measurement models such as Rasch models [9] have been used to explore the factor structure of the FACT-G. Rasch models allow estimates of item location ("difficulty") and person measures ("ability") to be made along postulated latent traits, such as for instance, pain, and physical and mental health. The strength of these models is that these parameter estimates are independent of the sample and questionnaires used. A recent study by Dapueto et al. [10] of version 4 of the FACT-G using Rasch analyses on responses derived from a Spanish-speaking (Uruguayan) cancer patient population demonstrated that with the exception of one item from the Social & Family Well-being scale ("I am satisfied with my sex life") and one item from the Emotional Well-being scale ("I am satisfied with how I am coping with my illness"), there were four unidimensional structures corresponding to the domains of the FACT-G (the relationship with doctor scale has been removed from more recent versions of the instrument). However, no Rasch analysis of the overall FACT-G total scale was performed, and in particular, the authors did not use Rasch models to explore the dimensionality of the FACT-G.

The concept of dimensionality and the concomitant of item fit (i.e. whether items fit the unidimensional Rasch construct) are important considerations in Rasch models, since only scales with items which fit the model give rise to interval based measures. This in turn allows meaningful interpretations of changes in scores [11,12]. Removal of misfitting items is advocated and often undertaken to improve the measurement properties of instruments [13]. However little is known of the relationship between (mis)fit and clinical utility. Studies have suggested that misfitting items can be removed without impact on the measurement properties of instruments [11,14,15], but few studies have explored the impact on clinical utility. Those few studies that have, have found that where misfitting items are removed and the original study analysis repeated with the reduced questionnaire, the overall results and conclusions of the studies did not change (i.e. no significant impact on clinical utility was found)[16].

The aim of this study was to investigate the dimensionality or factor structure and measurement properties of the FACT-G using both traditional psychometric analyses, such as Factor Analysis, and item and sample independent models, such as Rasch Models. Furthermore, a Rasch analysis of the FACT-G total was also performed to assess whether the entire scale formed a unidimensional construct. The relationship between item (mis)fit and clinical utility was explored by assessing the impact of the removal of misfitting items from the instrument on the ability of the scales to detect differences in scores between different patient groups in a clinical trial.

## Methods

### Patients

The patient data used for the Factor and Rasch analysis of the FACT-G were collated from two studies, one published [3] and another unpublished, which have been carried out by the Cancer Research UK, Psychosocial and Clinical Practice Research Group (St. James's University Hospital, Leeds). In the first study, 265 patients completed the paper version of FACT-G as an outcome measure in a randomised trial investigating the effects of using regular QOL measurement in oncology practice. Patients completed the FACT-G on 4 occasions: baseline, after 3 outpatient consultations, at 4 and 6 months.

In the second study, one group of patients completed an electronic version of the FACT-G on a standalone computer with a touchscreen monitor (n = 200). The aim of the study was a comparison of a number of quality of life instruments.

The studies received ethical approval from the local ethics committee of the Leeds Teaching Hospitals NHS Trust (UK).

### Instrument

The FACT-G version 4 consists of four subscales, Physical Well-being (PWB), Social & Family Well-being (SFWB), Emotional Well-being (EWB), and Functional Well-being (FWB). These are rated on a five-point Likert scale (i.e. "Not at all", "A little bit", "Somewhat", "Quite a bit", "Very much"). The scale scores are derived by summing the raw scores, which range from 0 to 28 (or 0 to 24 for Emotional Well-Being). Scores from the Physical and Emotional Well-being scales (with the exception of one item) are reversed. A total score is derived by summing the scale scores from all four subscales (range 0 – 108). Higher subscale scores indicate better health, functioning, or well-being. The timescale for the FACT-G is the past 7 days. Missing items were treated according to the guidelines of the questionnaire developers, which involves prorating scores, i.e. calculating the mean for completed items for each subscale containing missing data (where there is a 50% or greater response) and substituting this for the missing data.

### Statistical methodology

#### Traditional psychometrics

##### Reliability and factor analysis

In addition to means and standard deviations of the scale scores, the internal consistency of each domain was assessed using Cronbach's alpha. A principal components analysis was performed on the raw scores, and the factor structure rotated using orthogonal rotations (varimax). Only factor loadings above 0.50 were considered as indicative of item loading.

##### Rasch analysis

Rasch models [9] are latent trait models which model a probabilistic relationship between the level of latent trait (commonly referred to as person "ability" or "measure") and the items used for measurement (item "difficulty" or "location"). Both person ability and item location (estimated in terms of log-odds or "logits") are located along the same continuum. The estimation procedure provides person ability estimates which are independent of the items employed in the assessment, and conversely estimates item locations independently from the sample of test users (or patients) employed.

The data were analysed with *Winsteps *software [17] using the Partial Credit Model for polytomous data [18]. Item locations and person measures were derived for the each of the four FACT-G scales.

Three important criteria for Rasch models were investigated, namely, unidimensionality, item fit and item invariance.

#### Dimensionality

Unidimensionality concerns whether the data form a single factor [19] and can be used to assess whether the single latent trait explains all the variance in the data. Unidimensionality of each scale was evaluated with principal components analyses (PCA) of the residuals once the initial latent trait (i.e. the "Rasch" factor) has been extracted [17]. The following criteria were used to determine whether additional factors were present in the residuals: 1). a cutoff of 60% of the variance explained by the Rasch factor; and 2) eigenvalues smaller than 3 and the percentage variance explained by the first contrast of less than 5% [17]. However, recent studies have demonstrated that these measures might not be sufficient to determine multidimensionality [20].

Therefore, in addition to these criteria a method recommended by Smith [21] was employed to identify any potential multidimensionality: Item parameters for misfitting items were estimated with the entire scale, as well as independently for just those misfitting items. These two estimates for each misfitting item were then subtracted from each other and an average, or shift constant [21] calculated. Person measures were calculated for the entire scale (including misfitting items), as well as by using the misfitting items alone. The latter are then weighted using the shift constant (added to the person measures estimated by the misfit items alone) and independent t-tests performed for each pair of person measures. The percentage of tests falling outside the 95% confidence interval, ± 1.96, may then be evaluated. Since within the Rasch model person measures should agree within a certain degree of error irrespective of the subset of items used in the estimation process, any significant number of tests outside this interval will indicate the presence of multidimensionality.

#### Item fit and location

The item fit to the Rasch model is commonly measured by the mean-square residual fit statistic [19]. Two commonly employed fit statistics to assess item fit are the weighted mean square or infit statistic, and the unweighted mean square or outfit statistics. The outfit statistic is sensitive to anomalous outliers for either person or item parameters, whereas the infit statistic is sensitive to residuals close to the estimated person abilities. Fit statistics for items have an expected value of 1.0, and can range from 0 to infinity. Deviations in excess of the expected value can be interpreted as 'noise' or lack of fit between the items and the model, whereas values significantly lower than the expected value can be interpreted as item redundancy or overlap.

Item fit was assessed for the four subscales (Physical, Social & Family, Emotional, and Functional Well-being), as well as the FACT-G total. Fit was evaluated against a range of 0.70 – 1.30 for infit (weighted) mean squares [22], as well as outfit (unweighted) mean squares greater than 1.4. Any misfitting items (fit > 1.30) were removed from the individual scales and the Rasch analysis re-run. This iterative process was continued until no further misfit was observed. The item location was determined for the final iterative process for those items falling within the fit range (< 1.30) once misfitting items had been removed. The fit and item locations were also recorded for misfitting items.

#### Item invariance (Differential Item Functioning)

Item invariance refers to the fact that the estimated item location parameters should not be dependent on the sample used to derive the estimates. Rasch models require the item estimation to be independent of the subgroups of individuals completing the questionnaires. In other words, item parameters should be invariant across populations [23]. Items not demonstrating invariance are commonly referred as exhibiting differential item functioning (DIF) or item bias. Identification of differential item functioning (DIF) allows comparisons and evaluations to be made of whether items are functioning equivalently across important categories, such as diagnosis, extent of disease. Item invariance can be assessed by producing independent estimates of item location using subgroups of individuals (e.g. groups defined by gender, age group, diagnosis etc.).

As two different samples were used for the Rasch and Factor Analysis and as the data were derived through different modes of administration, differential item functioning analysis was used to determine whether item invariance held between the item parameters estimated from the two samples. Item invariance was derived by holding item location parameters constant while person measures were estimated separately for each age group [17]. This was then evaluated using a paired t-test. Item invariance was evaluated using a contrast between item difficulties of 0.5 logits or greater and a Bonferroni adjustment was applied to control for any effects due to multiple testing [22]. Therefore contrasts between parameters were evaluated at a level of significance (α) of 0.01 (t > 2.56).

As the outcome of this analysis would determine whether the data from the two samples could be pooled for the Factor and Rasch analyses, the differential item functioning analysis was carried out first.

#### Analysis of clinical significance of removal of misfitting items

The data used for this analysis was derived from a randomised control trial exploring the impact of measuring and using health-related quality of life (HRQoL) on doctor-patient communication and patient well-being [3]. Patients were randomly allocated to one of three arms depending on whether they regularly completed the intervention HRQoL questionnaire on a touchscreen computer prior to each clinic visit, whether these results were fed back to their physicians or receiving standard care [3]. Patients completed the FACT-G as an outcome measure at home at four time points.

The data used in this analysis are derived from the first two time points: baseline and after 3 outpatient consultations (approximately 2–3 months after baseline completion). The 3 study arms were compared in terms of changes in FACT-G over time (scores at time 1 minus baseline scores) using univariate analyses of variance and regression analysis. The dependent variable was the change in FACT-G domains and total scores. Study arm, performance status, gender and diagnosis were entered as fixed factors, and baseline FACT-G score per domain, age and time on study as covariates.

New scale scores were derived for each FACT-G subscale (PWB, SFWB, EWB & FWB), as well as the total FACT-G score by removing any misfitting items identified in the Rasch analysis and each subscale was rescored. The above analyses were carried out for the original and rescored FACT-G subscale scores and total score.

In addition to this analysis, the impact of removing misfitting items was also assessed through its influence on effect size [24]. Effect sizes were calculated by subtracting scores at time 1 from the baseline score and dividing this by the standard deviation of the baseline score for each subscale and rescored subscale, as well as the FACT-G total.

## Results

### Participants

A total of 465 patients completed the FACT-G, however demographic details were available for 461 patients, 323 females (average age 55.7 years, s.d. 12.4) and 138 males (average age 60.8, s.d. 13.0). Fewer than 5% of patients attending the outpatient clinics where the samples were collected were from ethnic minority communities. Table 1 gives a breakdown of diagnoses and scores from the FACT-G.

**Table 1:** Diagnosis by gender and age and mean scores (standard deviations) of the FACT-G domains

**Patient Characteristics**		
Age, years (mean ± S.D.)		
Female (n = 323)	55.7 ± 12.4	
Males (n = 138)	60.8 ± 13.0	
		
**Diagnosis**	**Count**	**%**
Breast	99	21.57
Colorectal	72	15.69
Gastrointestinal	27	5.88
Genitourinary	132	28.76
Lung	22	4.79
Melanoma	21	4.58
Renal	44	9.59
Sarcoma	19	4.14
Other	23	5.01
		
**FACT-G Scores**	**Mean**	**s.d.**
Physical (PWB)	26.28	6.06
Social & Family (SFWB)	16.08	15.15
Emotional (EWB)	22.96	4.87
Functional (FWB)	23.71	6.43
Total	89.78	20.60

### Item invariance (Differential Item Functioning)

No significant item invariance or bias was found for the items from the two samples for the subscales and FACT-G total scale. All contrasts for the subscales and FACT-G total score fell below the 0.5 logit criterion (p > 0.01). Therefore, there were no significant differences in the FACT-G item parameters derived from the two forms of administration, and the combined data from the touchscreen administration and baseline measurement in the randomised trial were used for the Factor and Rasch analysis.

### Reliability and factor analysis

Internal reliability of the four subscales was very good with Cronbach's alpha ranging between 0.81 and 0.85 for Physical and Functional Well-being, respectively, and between 0.78 and 0.72 for the Social & Family and Emotional Well-being scales, respectively.

The rotated component matrix is shown in Table 2. A total of 4 factors with eigenvalues significantly greater than 1.2 were extracted. These collectively represented almost 55% of the variance with 27%, 14%, 9% and 5% of variance explained respectively by each factor. The factors identified corresponded largely to the FACT-G subscales. Factor 1 corresponded to the Physical Well-being (PWB) scale with four items from the Functional Well-being (FWB) scale also loading (not significantly) onto this factor (""ability to work, "sleeping well", "enjoy things usually done for fun" and "contentment"). Factor 2 corresponded to most of the Functional Well-being scale (except for item FWB5, "Sleeping well"), as well as one item from the Social & Family Well-being (SFWB) scale, concerning family acceptance about the illness, and one item from the Emotional Well-being (EWB) scale (EWB2, "I am satisfied with how I am coping with my illness"). Factor 3 corresponded to four of the seven items from the Social & Family Well-being scale, namely items SFWB1, SFWB2, SFWB3 concerning support from family and friends and SFWB5 family communication. Neither items regarding closeness to partner and satisfaction with sex life loaded onto this or any other factor. Factor 4 corresponded to most of the Emotional Well-being scale, except for item 2 ("I am satisfied with how I am coping with my illness") which loaded onto Factor 2.

**Table 2:** Rotated Factor Loadings for the FACT-G

	**Component**			
	**1**	**2**	**3**	**4**
**PWB1 **– Lack of energy	**.72**	.13	-.10	.01
**PWB2 **– Nausea	**.61**	.02	.08	.03
**PWB3 **– Meeting needs of family	**.70**	.25	-.12	.11
**PWB4 **– Pain	**.53**	.07	-.12	.36
**PWB5 **– Side effects of treatment	**.57**	.07	-.05	.20
**PWB6 **– Feel ill	**.80**	.08	-.04	.16
**PWB7 **– Forced to spend time in bed	**.76**	.04	.10	.02
**SFWB1 **– Feel close to friends	.09	.12	**.86**	.04
**SFWB2 **– Emotional support from family	-.21	.30	**.74**	.06
**SFWB3 **– Get support from friends	.10	.18	**.87**	.00
**SFWB4 **– Family has accepted illness	-.12	**.53**	.46	.28
**SFWB5 **– Satisfactory family communication about illness	-.12	.49	**.64**	.19
**SFWB6 **– Feel close to partner	-.11	.40	.33	-.06
**SFWB7 **– Satisfied with sex life	.18	.43	.24	-.11
**EWB1 **– Feel sad	.24	.10	.08	**.60**
**EWB2 **– Satisfied with how coping with illness	-.04	**.62**	.04	.10
**EWB3 **– Losing hope in fight against illness	.10	.03	.09	**.58**
**EWB4 **– Feel nervous	.13	.06	.00	**.69**
**EWB5 **– Worry about dying	-.01	.14	.08	**.82**
**EWB6 **– Worry condition will get worse	.08	.03	-.04	**.76**
**FWB1 **– Able to work	.43	**.66**	.00	-.01
**FWB2 **– Work is fulfilling	.35	**.66**	.11	.02
**FWB3 **– Able to enjoy life	.37	**.69**	.28	.15
**FWB4 **– Accepted illness	-.08	**.64**	.20	.21
**FWB5 **– Sleeping well	.41	.38	.28	.15
**FWB6 **– Enjoy things usually done for fun	.44	**.62**	.31	.02
**FWB7 **– Content with quality of life	.44	**.63**	.14	.15

*PWB: Physical Well-being; SFWB: Social & Family Well-being: EWB: Emotional Well-being; FWB: Functional Well-being.

### Rasch analysis

#### Dimensionality

The results of the principal components analysis (PCA) of the residuals of the scales suggested that no additional structures were present in all of the FACT-G scales.

For the Physical Well-being scale 81.5% of the variance was explained by the measures, whereas only 4.7% of the unexplained variance was accounted for by the first contrast (eigenvalue of 1.8). For Social & Family Well-being, Emotional Well-being and Functional Well-being respectively the variance explained by the Rasch model amounted to 78.9%, 76.1%, and 72.3%. The unexplained variance amounted to 6.2%, 7.1% and 8.0% (2.0, 1.8 and 2.0 eigenvalues), respectively.

The additional analysis revealed no significant number of person measure pairs falling outside the 95% confidence for the Emotional Well-being scale (<1% interval or 3/451 pairs) and Functional Well-being scale (5.3%, 24/451) demonstrating that these scales did not demonstrate any multidimensionality. However, a significant number of pairs for the Social & Family Well-being scale did exceed the 5% criterion (37.25%, 168/451) indicating that multidimensionality is present in this scale. This analysis could not be carried out on the Physical Well-being scale as only one item demonstrated misfit, which did not allow item and person parameters to be estimated.

#### Item fit and location

The final location measures and fit statistics for all the FACT-G scales are provided in Table 3. One item was identified as misfitting from the analysis of the Physical Well-being, namely PWB1 ("I have a lack of energy"). In addition, one item from this scale, namely PWB6 ("I feel ill") displayed redundancy. For the Social & Family Well-being scale two items demonstrated misfit namely items SWB6 ("I feel close to my partner (or the person who is my main support") and SWB7 ("I am satisfied with my sex life"). One item from this scale demonstrated some redundancy, but only by the outfit fit statistics (SFWB2, "I get emotional support from my family"), which were not used to identify misfit. Two items from the Emotional Well-being scale demonstrated misfit, (EWB2 "I am satisfied with how I am coping with my illness") and EWB6 ("I worry that my condition will get worse"). No items demonstrated redundancy. Two items from the Functional Well-being scale, i.e. FWB4 ("I have accepted my illness") and FWB5 ("I am sleeping well") also demonstrated misfit. All items from the scales demonstrated good fit when the misfitting items had been removed (Table 3). In addition, the recalibrated item parameters fell within ± 0.5 logit of the initial item location for each item.

**Table 3:** Item location and fit for FACT-G

			**Infit**	**Infit**	**Outfit**	**Outfit**
**Items**	**Person Measure**	S.E.	MNSQ	ZSTD	MNSQ	ZSTD
**PWB1**	-0.31	0.06	**2.00**	9.90	**1.95**	9.90
**PWB2**	0.13	0.06	0.92	-1.00	0.95	-0.50
**PWB3**	-0.07	0.06	1.27	3.50	1.17	2.00
**PWB4**	0.06	0.06	1.27	3.50	1.29	3.20
**PWB5**	-0.12	0.06	1.22	3.10	1.19	2.60
**PWB6**	-0.08	0.06	*0.58*	-6.90	*0.55*	-6.90
**PWB7**	0.09	0.05	0.75	-3.50	0.69	-3.30
						
**SWB1**	0.66	0.08	1.19	2.20	1.14	0.77
**SWB2**	-0.28	0.09	0.81	-1.80	*0.66*	-2.60
**SWB3**	0.20	0.08	0.91	-1.00	0.87	-1.50
**SWB4**	-0.30	0.08	1.13	2.20	1.20	1.90
**SWB5**	-0.28	0.09	0.85	-1.60	0.83	-1.40
**SWB6**	-0.62	0.09	**1.37**	2.30	**1.63**	2.30
**SWB7**	2.30	0.07	**1.57**	5.00	**2.63**	7.40
						
**EWB1**	-0.07	0.07	1.00	0.00	0.95	-0.70
**EWB2**	-0.78	0.06	**2.10**	9.90	**2.36**	9.90
**EWB3**	0.23	0.06	1.14	1.40	1.41	2.20
**EWB4**	-0.10	0.07	0.81	-2.80	0.80	-3.00
**EWB5**	-0.05	0.06	1.06	0.80	0.97	-0.40
**EWB6**	-0.34	0.06	**1.49**	6.40	**1.42**	5.30
						
**FWB1**	0.60	0.06	1.30	4.00	1.37	4.40
**FWB2**	0.45	0.06	1.10	1.50	1.05	0.60
**FWB3**	-1.04	0.07	0.77	-3.70	0.76	-3.70
**FWB4**	-1.03	0.06	**1.38**	4.70	**1.59**	5.40
**FWB5**	-0.20	0.06	**1.58**	7.50	**1.74**	8.40
**FWB6**	0.00	0.06	0.87	-1.90	0.87	-1.80
**FWB7**	-0.01	0.06	0.92	-1.10	0.89	-1.40

*Infit/Outfit > 1.3 is highlighted in bold; Infit/Outfit < 0.70 is highlighted in italics.

** Table shows final item locations for scales when misfitting items have been removed, as well as initial fit statistics & parameters for those misfitting items.

The range of locations for items from the Physical Well-being scale was narrow (-0.31 – 0.13), for both the Emotional Well-being and Social & Family Well-being scale this range was moderately greater (-0.34 – 0.23, and -0.30 – 0.66 respectively). Functional Well-being scale covered the greatest item range (-1.04 – 0.60).

#### Rasch Analysis of the FACT-G total

For the FACT-G total scale an additional factor was extracted with 7.0 eigenvalues accounting for 8% of the unexplained variance with the Rasch factor accounting for 69.5%. However, there were no significant numbers of person measures falling outside the 95% confidence interval (4.21%, 19/451) demonstrating that the scale was unidimensional.

The FACT-G total (results not shown) showed two misfitting items (PWB1 and SWB7). Infit mean squares for all remaining items fell below the 1.3 criterion. Finally, the range of item locations for the FACT-G total scale was fairly limited (-0.91 – 0.50).

#### Analysis of clinical significance of removal of misfitting items

There were significant differences between study arms for three of the FACT-G subscales and the FACT-G total for the original, full scales (Table 4).

**Table 4:** Univariate analysis of variance, comparing randomised trial results with original FACT-G total scores and scales scores and abbreviated scales following removal of misfitting items

	**Original scales**			**Misfitting items removed**		
	**F statistics**	**P value**	**Effect size**	**F statistics**	**P value**	**Effect size**

**FACT-G**	5.32	0.006	0.16	3.00	0.05	0.18
**PWB**	4.45	0.013	0.16	3.91	0.022	0.14
**FWB**	3.79	0.024	0.16	6.39	0.002	0.14
**EWB**	4.84	0.009	0.24	4.78	0.01	0.23
**SFWB**	<1	>0.1	0.10	<1	>0.1	0.20

The results from the analysis were similar for the scales and total scores following removal of misfitting items. It should be noted that the values of F statistics were lower for the abbreviated scales, thus suggesting some loss of statistical power to detect differences. The one exception was the FWB, where the removal of misfitting items led to an increased value of the F statistic. There were no significant differences for both the original SFWB and revised SFWB (F < 1).

The effect sizes for each subscale and FACT-G total score did not differ when misfitting items were removed with the exception of the Social & Family Well-being scale where effect size improved when the misfitting items were discarded (Table 4). Furthermore, only the effects sizes observed for both EWB scales and the rescored SFWB scale could be considered as being minimally important differences [24].

## Discussion

This study described a traditional factor analysis and Rasch analysis, which were carried out on the FACT-G [4]. The results from the initial (rotated) factor analysis (principal components analysis) demonstrated a four-factor structure, which largely corresponded to the FACT-G subscales with high levels of internal consistency.

The results of the subsequent Rasch analysis of each subscale showed that all subscales had items which misfit, although the majority did not demonstrate any redundant items. The subscales were unidimensional, the only exception to this was the Social and Family Well-being scale where 37% of the paired person measures fell outside the 95% confidence interval therefore demonstrating multidimensionality in the scale. This suggests that the Social and Family Well-being scale is perhaps a two-factor scale with factors corresponding to family concerns, in particular emotional support from family, and family communication and acceptance of the illness (items SFWB1 to SFWB5), and another factor relating, primarily to close personal relationships (SFWB6 and SFWB7).

Comparisons between the results of the Factor Analysis and the Rasch analysis should be carried out with caution as the fundamental aims of each method differ with the aims of principal components factor analysis being to identify factors within a correlation matrix, whereas for Rasch analysis the object is to determine whether multidimensionality exists in the residuals once the unidimensional structure has been removed from the analysis [17]. Nevertheless, in general, the item misfit observed from the Rasch analysis in particular for the Social & Family Well-being and Emotional Well-being scales corresponded broadly to the results from the Factor Analysis.

Removing items from the questionnaires (scales) which do not fit the Rasch model may improve the measurement properties of the instrument. It is important also to investigate whether these theoretical improvements may change the ability of the instrument to detect group or differences in clinical situations over time. Our results of applying this investigation to the analysis of a randomised trial where FACT-G was an outcome measure, suggest that removing the misfitting items had no impact on item locations, nor on the ability of the revised instruments to detect significant changes in scores between patient groups. Furthermore, removing misfitting items did not affect the effect sizes for the majority of subscales and the FACT-G total, although an improved effect size was observed for Social & Family Well-being.

These findings have to be replicated in other studies. If confirmed, they may have important implications for how misfitting items should be treated. Clearly a balance needs to be struck here between clinical utility and measurement properties of the instrument. Items in scales may very well exhibit misfit (although still retain "face validity"), and removal of misfitting items may have no impact on the measurement properties of the instrument [14,15]. However, the results from this study suggests at best that removal of misfitting items does not affect clinical utility for the scales, although the statistical power to detect differences may be reduced for the revised instruments or scales, with the notable exception of FWB scale where the power appeared to be increased.

Caution should perhaps be exercised when identifying misfitting items. Some concerns have been expressed in the Rasch literature about the ability of a single residual-based fit statistic to correctly identify misfit across a range of assessment scenarios and instruments [25]. Therefore, the removal of these items should be assessed against the impact on clinical utility. This could entail evaluating measurement properties not only against fit statistics, but also against external clinical criteria. For instance, in our earlier work we evaluated the impact of removal of misfitting items from the Hospital Anxiety & Depression Scale (HADS) in a cancer population Smith et al. [16]. The study found that the sensitivity and specificity of the instrument as measured against a clinical interview was not affected by the elimination of misfitting items.

Although both this study and the results from Dapueto et al. [10] identified the same misfitting items (fit > 1.3) from the Social & family Well-being and Emotional Well-being Scales (SFWB7: "I am satisfied with my sex life"; EWB2: "I am satisfied with how I'm coping with my illness"), results from this study indicated an additional misfitting item in these two scales, as well as misfit in the other two scales. This was not simply due to any differences in the range of fit statistics employed, since the fit statistics for the remaining items from the Dapeuto study fell within the 0.7 – 1.3 range employed in this study. Differences may have arisen due to differences in the English and Spanish translation of the FACT-G. Differential item functioning has, in particular, been observed between different language versions of other HRQoL questionnaires (e.g. the EORTC QLQ-C30, [26]). In addition to this, a significant proportion of the patients (68%) in the Dapueto study required assistance when completing the questionnaire. Although the researchers concluded that little relevant difference was found in terms of the internal reliability of the scales between those questionnaires which were self-administered and those which were read to patients, the problem remains that different forms of administration may have affected the results and could explain the discrepancies between these two studies.

In a separate Rasch analysis it was also demonstrated that the FACT-G total scale was unidimensional once two misfitting items had been removed, suggesting that the scale may be used as a summary index, indicating an overall level of quality of life or well-being. This may facilitate the interpretation of well-being scores within clinical practice, as an adjunct to the scores derived for each subscale, and may also potentially facilitate the use and interpretation of FACT-G scores when used as an outcome measure in clinical trials.

The potential limitations of this study are the fact that although the diagnosis of patients was heterogeneous just over 50% of the patients had either breast or genitourinary cancer reflecting a greater proportion of women who participated in the studies. Furthermore, no additional clinical data was available of stage or extent of disease to evaluate whether the analysis held across disparate clinical subgroups. The analysis should perhaps be replicated with a larger sample size, although studies have demonstrated that Rasch models are able to produce robust estimates for item locations and fit statistics for sample sizes of 100 [27].

In summary, the Rasch analysis of the FACT-G subscales demonstrated that three of the four subscales and the FACT-G scale were unidimensional, although all subscales and the total scale contained misfitting items. Some caution needs perhaps to be exercised in interpreting the results from the Social & Family Well-being scale, particularly when employing a single score as an index of a clinically meaningful difference [5], since if the subscales do not represent a single underlying construct it becomes difficult to draw valid conclusions from a change in scores [12].

Therefore, future work needs to be conducted utilising larger sample sizes to determine whether item misfit holds for all scales, and whether these are observed in clinical subgroups (e.g. different diagnoses and stage of disease). In general, the relationship between item fit and clinical utility should also be explored in more detail and in particular what the impact of misfit is on the clinical utility of the instrument. Furthermore, the multidimensionality of the Social & Family Well-being scale and the potential existence of two subscales ("Family" and "Close personal" relationships) also needs to be investigated.

## Conclusion

Both the Factor and Rasch analyses demonstrated that all the FACT-G scales and total scale were unidimensional with the exception of the Social & Family Well-being scale. The Rasch analysis revealed misfitting items for each subscale. Removal of the misfitting items did not impact on clinical utility of the scales.

## Competing interests

The author(s) declare that they have no competing interests.

## Authors' contributions

ABS undertook the analysis of the data and writing of the manuscript. GV contributed to the data collection, analysis of clinical utility, writing and critical reviewing of the manuscript. EPW and PJS contributed to the preparation and writing of the manuscript.
